# A comparative study to assess the use of chromium in type 2 diabetes mellitus

**DOI:** 10.25122/jml-2023-0081

**Published:** 2023-08

**Authors:** Fatima Alkhalidi

**Affiliations:** 1 Department of Community Medicine, College of Medicine, University of Al-Qadisiyah, AL-Qadisiyah, Iraq

**Keywords:** Chromium, diabetes, type II diabetes mellitus, chromium supplementation

## Abstract

Diabetes mellitus is a prevalent endocrine disorder characterized by elevated blood glucose levels, often resulting in complications affecting multiple organs, such as retinopathy, nephropathy, and neuropathy. Among potential interventions, certain micronutrients, like chromium, have the potential to improve glycemic management. The potential of chromium to mitigate insulin resistance and enhance insulin sensitivity through cellular receptors underscores its significance. Conversely, insufficient dietary chromium intake could contribute to diabetes development. This research aimed to evaluate the impact of chromium supplementation among individuals with diabetes. In a single-blind randomized clinical trial, participants aged 40 to 60 years with uncontrolled diabetes were divided into two groups. The intervention group received a daily chromium supplement of 200 mcg and their regular diabetes medication regimen, while the control group received only medication. The follow-up period spanned four months, during which fasting blood sugar, HbA1c levels, and lipid profiles were assessed for both groups, followed by a comparative analysis. Patients had a mean age of 52.3±6.3 years. Males constituted only 47.5% of participants, and women were 52.5%. The initial HbA1c level at the start of the study for individuals receiving chromium was 10.4±2.4. Following the follow-up period, the average HbA1c level decreased significantly to 7.2±1.7, showing a statistically significant difference. Furthermore, there was a significant reduction in the mean fasting blood sugar levels, approaching normal levels. These results suggest a beneficial role of chromium supplementation in managing type 2 diabetes mellitus, contributing to improved glycemic control.

## INTRODUCTION

Diabetes mellitus is a prevalent endocrine disorder characterized by elevated blood glucose levels and accompanied by complications affecting diverse organs, such as retinopathy, nephropathy, and neuropathy [1]. Prediabetes, a group of conditions including impaired glucose tolerance and metabolic syndrome, signals an increased risk of diabetes [2]. Prediabetes and diabetes can increase the economic burden by increasing treatment costs and causing individuals to withdraw from work due to the extended treatment duration imposed by its complications [3]. The management of diabetes requires a rigorous medication regimen, dietary adjustments, and lifestyle changes, all aimed at mitigating health risks and minimizing the long-term impacts of associated complications [4].

Both macro and micronutrients are crucial for sustaining various physiological functions in the human body. While micronutrients are required in small quantities, they have key roles in many body systems. These micronutrients facilitate diverse pathways, including enzyme regulation, cellular actions, and hemostasis [5]. Micronutrients include four categories: microelements, trace materials, vitamins, and organic acids. Phosphorous, chloride, magnesium, calcium, iron, potassium, and sodium are major macro elements. On the other hand, trace elements like chromium, cobalt, copper, iodine, and zinc play significant roles in insulin activity, enhancing insulin sensitivity within cells [6]. Consequently, there is a particular focus on investigating the role of trace elements, given their relevance to the development of type 2 diabetes mellitus (T2DM). Studies observed alterations in the actions and levels of trace elements in conditions associated with diabetes mellitus, underscoring the need to study these elements in detail [7].

Many micronutrients could have positive protective effects in controlling blood sugar. Chromium, for instance, can reduce insulin resistance and enhance insulin sensitivity via cell receptors [8]. Conversely, the absence of adequate chromium in the diet could contribute to the development of diabetes [9]. Chromium is essential for facilitating the metabolism of lipids, proteins, and carbohydrates [1]. Nevertheless, the role of chromium in diabetes onset and treatment remains uncertain due to inconclusive findings regarding its precise impact on insulin sensitivity and actions from existing studies [10]. The daily recommended chromium supplement intake for both males and females falls within the range of 25-35 mg/day, as advised by national health institutions. Chromium supplementation is available in different forms, such as trivalent chromium chloride, commonly found in everyday foods like broccoli, green beans, and whole grains worldwide [5]. Another variation is chromium picolinate, a synthetic compound [1]. Chromium is crucial in various critical physiological processes within the body, notably in glucose metabolism. It achieves this by activating insulin receptors through chromodulin oligopeptides, leading to an enhanced transmission of insulin signals and heightened sensitivities [8]. When the body is deficient in chromium, it can lead to various metabolic effects, including impaired glucose tolerance, elevated levels of circulating insulin, hyperglycemia, and growth impairments [9]. The pivotal role of chromium emerged when administering fluids and nutrition to hospitalized patients inadvertently triggered diabetes development; nevertheless, introducing chromium-rich nutrition significantly decreased diabetes prevalence [11]. Following this discovery, researchers initiated numerous studies on chromium nutrition and its implications in diabetes management, as well as its broader effects on cellular function. Their focus centered on chromium's role in regulating blood glucose [5].

Chromium is stored within cells when it binds to specific small-sized proteins. This action is initiated when insulin binds to its receptors, and chromium helps transmit insulin messages before being excreted both from cells and the body as a whole. This mechanism results in a decrease in chromium levels, especially within glucose-dependent pathways. Consequently, elevated blood glucose prompts heightened insulin action, receptor binding, and a subsequent loss of chromium [12]. Numerous studies highlight improvements in glucose levels post-chromium supplementation. One such study from China reported enhanced insulin sensitivity, decreased insulin levels, reduced lipid profiles, and decreased HbA1c levels following management. Furthermore, twelve controlled studies on chromium treatment showed enhanced blood sugar control and reduced lipid profiles among healthy middle-aged participants [12]. However, three controlled studies reported no significant effect of chromium supplementation on glucose levels in patients with diabetes mellitus [13]. These studies indicate that supplementing with dietary chromium rich in this micronutrient enhances insulin actions, known as the glucose tolerance factor [14]. Thus, the focus of this study was to evaluate the impact of chromium supplementation on patients with diabetes.

## MATERIAL AND METHODS

### Study design and participants

A single-blind randomized clinical trial was conducted, enrolling patients aged 40 to 60 years to investigate the impact of chromium intake on the control of type two diabetes mellitus. Patients with uncontrolled diabetes were randomly assigned into two groups. One group received a chromium supplement (200 mcg, BID) in addition to their regular diabetes medication regimen, while the other group relied exclusively on the standard drug treatment. The inclusion criteria for this study included patients diagnosed with uncontrolled type two diabetes mellitus. On the other hand, the exclusion criteria comprised individuals who declined participation, failed to return for follow-up visits, had serum creatinine concentration exceeding 2.0 mg/dL at baseline, reported a history of pancreatitis, recently underwent significant abdominal surgery, were pregnant or intended to become pregnant during the study, or had polycystic ovarian syndrome or irregular menses. The study extended over a three-month follow-up period during which fasting blood sugar, HbA1c levels, and lipid profiles were measured for both groups, facilitating subsequent comparisons. The study was conducted for six months at the Aldiwanyia Teaching Hospital/Diabetes Center in Iraq.

### Data collection and analysis

Sixty patients were recruited for the study, with 30 individuals allocated to each group. The allocation process was carried out using random numbers generated by a computer, utilizing the participants' record numbers from the clinic. Initial data collection included demographic information, anthropometric measurements, and blood investigation results, including Fasting Blood Sugar (FBS), HbA1c, and lipid profiles. Blood investigations were repeated at the end of the three-month follow-up period to assess changes. The study also evaluated the proportion of patients achieving glycemic control in each group.

### Blood sample collection

Ten milliliters of blood were collected from each patient. Five milliliters were used for the HbA1c assay in tubes containing EDTA. Four milliliters were collected for lipid profiles, and one milliliter was used for fasting blood sugar analysis. Glucose assay analysis was conducted immediately, while the remaining samples were centrifuged and stored at -21°C until measurement.

### Body mass index measurements (BMI)

BMI was calculated using the formula established by the World Health Organization: weight in kilograms divided by height in square meters. The BMI results were classified into different categories: underweight (BMI<18), normal weight (BMI 18-24.9), overweight (BMI 25-29.9), and obesity (BMI>30).

### Statistical analysis

Data were collected using Microsoft Excel and then analyzed using SPSS version 24. Categorical variables were presented as numbers and percentages and analyzed using the chi-square test. Continuous variables were reported as means and standard deviations. The analysis of continuous variables was conducted using both the student t-test and paired t-test. A p-value of less than 0.05 was considered statistically significant.

## RESULTS

**Figure 1:**
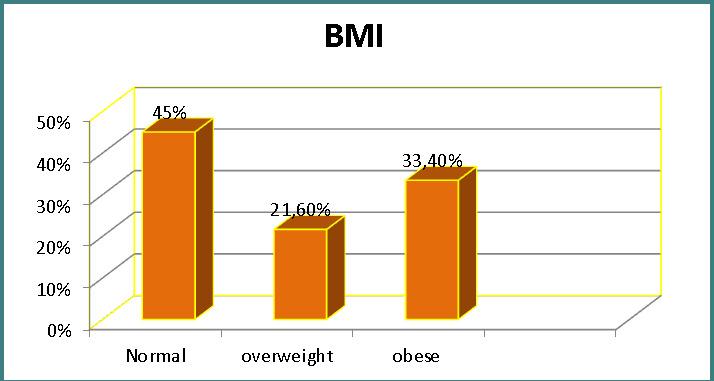
Distribution of patients according to BMI

Patients had a mean age of 52.3±6.3 years (range 40-60 years). Gender distribution revealed that males constituted 46.7% of the sample, while females accounted for 53.3%. According to residency, 76.6% of participants lived in urban areas, while 23.4% lived in rural regions (Table 1). Regarding body mass index (BMI) categories, 45% were classified as normal weight, 21.6% were overweight, and 33.4% were classified as obese (Figure 1). The overall mean BMI of patients was 27.1±0.4. Height and weight had mean values of 1.64±0.02 meters and 83.2±2.75 kilograms, respectively (Table 2).

**Table 1:** Demographic characteristics of the sample

Characteristics		No.	Percentage
**Gender**	Male	28	46.7%
	Female	32	53.3%
	Total	60	100%
**Residence**	Urban	46	76.6%
	Rural	14	23.4%
	Total	60	100%
**BMI**	Normal	27	45%
	Overweight	13	21.6%
	Obese	20	33.4%
	Total	60	

**Table 2:** Anthropometrics measurements

Variables	Mean ±SD
Height(m)	0.02±1.64
Weight (kg)	83.2±2.75
BMI (kg/m2)	0.4±27.1

After a three-month follow-up, the mean HbA1c level decreased significantly from 10.4±2.4 at baseline to 7.2±1.7 (p-value<0.001). Fasting blood sugar levels also significantly decreased, approaching normal levels (p-value<0.001).

Serum cholesterol levels decreased throughout the study period (p-value=0.002). Additionally, high-density lipoprotein (HDL) levels increased following chromium supplementation (p-value=0.006). Although the mean triglyceride level decreased, the change was not statistically significant (Table 3).

**Table 3:** Changes in HbA1c, FBS, and lipid profiles within the intervention group

Parameter	Baseline	After 3 months	p-value
HbA1C	10.4±2.4	1.7±7.2	0.001
FBS	20.9±154.6	19.1±128.3	0.001
BMI(kg/m2)	3.4±27.3	2.5±26.4	0.18
Cholesterol(mg/dL)	60.2±190.3	30.4±149.1	0.002
Triglyceride(mg/dL)	110.8±196.1	47.1±168.4	0.14
HDL(mg/dL)	9.6±36.5	10.3±42.4	0.006

Table 4 shows the metabolic changes in the control group regarding blood sugar and lipid profiles. Table 5 reveals the differences in different parameters between groups.

**Table 4:** Changes in HbA1c, FBS, and lipid profiles within the control group

Parameter	Baseline	After 3 months	p-value
HbA1C	10.8±1.3	2.4±9.6	0.07
FBS	22.1±164.6	20.5±154.3	0.18
BMI(kg/m2)	3.2±27.3	3.7±27.4	0.9
Cholesterol(mg/dL)	55.3±191.3	29.2±153.1	0.001
Triglyceride(mg/dL)	22.1±192.4	61.3±181.1	0.34
HDL(mg/dL)	10.2±35.5	12.1±39.2	0.14

**Table 5:** Comparison of HbA1c, FBS, and lipid profiles between intervention and control groups

Parameters		After 3 months	p-value
HbA1C	intervention	1.7±7.2	0.005
	control	2.4±9.2	
FBS	intervention	19.1±128.3	0.013
	control	20.5±141.3	
BMI (kg/m2)	intervention	2.5±26.4	0.2
	control	3.7±27.4	
Cholesterol (mg/dL)	intervention	30.4±149.1	0.6
	control	29.2±153.1	
Triglyceride (mg/dL)	intervention	47.1±168.4	0.3
	control	61.3±181.1	
HDL (mg/dL)	intervention	10.3±42.4	0.34
	control	12.1±39.2	

## DISCUSSION

Emerging evidence from multiple studies supports the positive impact of chromium on managing abnormal blood sugar levels in diabetes. It is believed to enhance insulin sensitivity in cells, facilitating insulin-cell interactions [15]. In addition to its effects on diabetes, chromium supplementation has been linked to reductions in blood pressure, cholesterol levels, and body weight, along with a decreased risk of metabolic syndrome [16]. Numerous research highlighted the role of chromium not only in managing type 2 diabetes and other metabolic conditions associated with its pathogenesis [17].

Studies have noted a consistent reduction in HbA1c and blood glucose levels following chromium supplementation, likely attributed to its enhancement of insulin sensitivity and improved glucose regulation [18]. While some studies have shown promising results for newly diagnosed type 2 diabetes patients, there is a range of findings in the literature [19].

Furthermore, chromium plays a significant role in lipid reduction. Multiple studies have demonstrated a notable decrease in triglyceride levels and an improvement in the risk associated with central obesity without any adverse impact on liver or renal function [18].

The study cohort had a mean age of 52.3±6.3 years, with 46.7% males and 53.3% females, aligning with similar studies [20, 21]. The three-month follow-up period adopted in the present study is consistent with the duration used in other investigations [1, 18]. The findings revealed a substantial reduction in HbA1c from 10.4±2.4 to 7.2±1.7 after three months of follow-up, along with a significant decrease in FBS levels (p-value<0.001). These outcomes correspond to other studies that highlight the beneficial effects of chromium on glycemic control [16, 18]. Positive outcomes were also noted in studies that evaluated various parameters and clinical results following three months of chromium supplementation [18]. Notably, one study demonstrated that administering chromium supplements over 3 months yielded beneficial effects among individuals with diabetes, both in control parameters and clinical enhancements [18]. The researchers concluded that chromium intake plays a positive role for patients with diabetes, leading to improved insulin sensitivity and reduced blood sugar levels following treatment.

Both this study and others have suggested that chromium improves insulin sensitivity, leading to decreased blood sugar levels in diabetes patients [18]. Similarly, research has demonstrated that chromium supplementation over four months reduces fasting blood sugar and HbA1c levels [22]. Studies exploring more extended supplementation periods, such as six months, have shown promising effects on insulin resistance reduction and improvements in BMI and fasting serum insulin [23]. Authors have concluded that chromium's potential to enhance insulin action, rather than insulin secretion, underscores its role in glycemic control for type 2 diabetes patients [17].

In contrast to the result of this research, a study reported no benefit from chromium supplementation in diabetic patients with different formulas [1]. Moreover, other studies concluded that chromium supplements have limited effectiveness, and there is little rationale to recommend their glycemic control use in patients with T2DM [24].

These discrepancies between studies may be due to differences in baseline insulin sensitivity (patients with insulin resistance respond better to chromium), quantity of chromium administered, study period, sample size, and type of diabetes mellitus [13].

The present study demonstrated a significant reduction in cholesterol serum levels over the study period (p-value=0.002). Additionally, the mean level of HDL increased following chromium supplementation (p-value=0.006). Although the mean level of triglycerides decreased, this difference did not reach statistical significance. These findings are consistent with the results of Ngala *et al*., where they observed improved glucose and lipid metabolism with increased plasma chromium concentration [20]. Conversely, Ismael San Mauro's study did not indicate that chromium supplement therapy in type 2 diabetes patients had a notable impact on HDL-c, LDL-c, and TG levels [25].

Plasma chromium levels can be influenced by various factors such as stress, diet, and exercise. Unfortunately, the potential impact of these confounding variables on chromium levels in our diabetes patient population could not be assessed.

## CONCLUSION

Chromium supplementation had positive effects in ameliorating the impact of type two diabetes mellitus. It has been identified as a beneficial factor in controlling blood sugar. In addition, it improved the lipid profile of diabetes patients with no effect on triglyceride levels.
